# A Core35S Promoter of Cauliflower Mosaic Virus Drives More Efficient Replication of Turnip Crinkle Virus

**DOI:** 10.3390/plants10081700

**Published:** 2021-08-18

**Authors:** Md Emran Ali, Sumyya Waliullah

**Affiliations:** Department of Plant Pathology, University of Georgia, Tifton, GA 31793, USA; Sumyya.Waliullah@uga.edu

**Keywords:** 35S promoter, turnip crinkle virus, virus replication, green fluorescent protein, virus infection

## Abstract

The 35S promoter with a duplicated enhancer (frequently referred to as 2X35S) is a strong dicotyledonous plant-specific promoter commonly used in generating transgenic plants to enable high-level expression of genes of interest. It is also used to drive the initiation of RNA virus replication from viral cDNA, with the consensus understanding that high levels of viral RNA production powered by 2X35S permit a more efficient initiation of virus replication. Here, we showed that the exact opposite is true. We found that, compared to the Core35S promoter, the 2X35S promoter-driven initiation of turnip crinkle virus (TCV) infection was delayed by at least 24 h. We first compared three versions of 35S promoter, namely 2X35S, 1X35S, and Core35S, for their ability to power the expression of a non-replicating green fluorescent protein (GFP) gene, and confirmed that 2X35S and Core35S correlated with the highest and lowest GFP expression, respectively. However, when inserted upstream of TCV cDNA, 2X35S-driven replication was not detected until 72 h post-inoculation (72 hpi) in inoculated leaves. By contrast, Core35S-driven replication was detected earlier at 48 hpi. A similar delay was also observed in systemically infected leaves (six versus four days post-inoculation). Combining our results, we hypothesized that the stronger 2X35S promoter might enable a higher accumulation of a TCV protein that became a repressor of TCV replication at higher cellular concentration. Extending from these results, we propose that the Core35S (or mini35S) promoter is likely a better choice for generating infectious cDNA clones of TCV.

## 1. Introduction

Promoters are DNA sequences that are receiving increased attention as one of the primary determinants of the initiation of transcription and modulation of levels and timing of gene expression [1,2]. It controls the accurate initiation of transcription by binding to the RNA polymerase II enzyme and transcribes a gene into RNA [3]. It is usually assumed to be the key *cis*-acting regulatory region that controls the transcription of the adjacent coding region(s) into messenger ribonucleic acid (mRNA), which is then directly translated into proteins [4]. Promoters can be identified as binding sites for *trans*-acting factors, “transcription factors”, which may cause activation or repression of transcription [4]. Among many plant promoters, the highly expressed cauliflower Mosaic Virus 35S (35S); a strong plant promoter, is widely used for gene regulation in many plants [2,5,6,7]. Having a duplicated enhancer (i.e., 2X35S); it enhances the level of promoter activity up to ten-fold in conjunction with other transcription factors [8,9]. Several studies have already reported that the 35S promoter with a duplicated enhancer is commonly used to drive the initiation of RNA virus replication from viral cDNA [10,11].

In this report, we investigated the strength of different versions of 35S promoter sequence for driving turnip crinkle virus (TCV) viral replicon. TCV (family *Tombusviridae*, genus *Carmovirus*) is a positive (+) sense single-stranded RNA virus of 4053 nt, which contains five major open reading frames [12]. The 5′ proximal p28 and its readthrough product p88 proteins are translated from genomic RNA, act together as RdRp and are involved in viral RNA replication. Following p28 and p88, two other replication dispensable smaller proteins p8 and p9 are expressed from 1.7 kb subgenomic RNA1 and are required for cell-to-cell movement. Finally, the 3′-proximal p38 is a coat protein (CP) translated from the 1.45-kb subgenomic RNA acts as an effective suppressor of RNA silencing [13,14].

Employing a strong promoter to maximize transgene expression is the conventional method of plant transformation; however, the expression effects might vary resulting from its interaction with other factors [15,16]. A previous study has shown that CaMV 19S promoter, a weaker promoter than the 35S is also able to regulate strong expression of the heterologous genes [17]. Another study focusing on the deletion analysis of the commelina yellow mottle virus (CoYMV) promoter revealed that the promoter had similar strength to the original CoYMV in stably transformed maize callus [18,19]. Similar results were obtained in the rice tungro bacilliform virus (RTBV) promoter analysis by deletion of the promoter sequences [20]. All of these studies did not directly evaluate the comparative effect of a weaker promoter to the stronger promoter on viral replication. Without any doubt, the well-studied 35S plant promoter architecture has been extensively analyzed; despite this, the nature of its enhancer’s effect on RNA viral replication remains to be unraveled. In this study, we revealed that the use of a weaker promoter helped enhance viral replicon which led to earlier symptom expression. To elucidate the underlying mechanism of this phenomenon, here we explored the use of the core35S promoter to achieve more efficient initiation of TCV virus replication than the two other versions of 35S promoter sequences (1X35S and 2X35S).

## 2. Results

### 2.1. Strength Variation of Three Variants of 35S Promoters by GFP Expression

**Figure 1:**
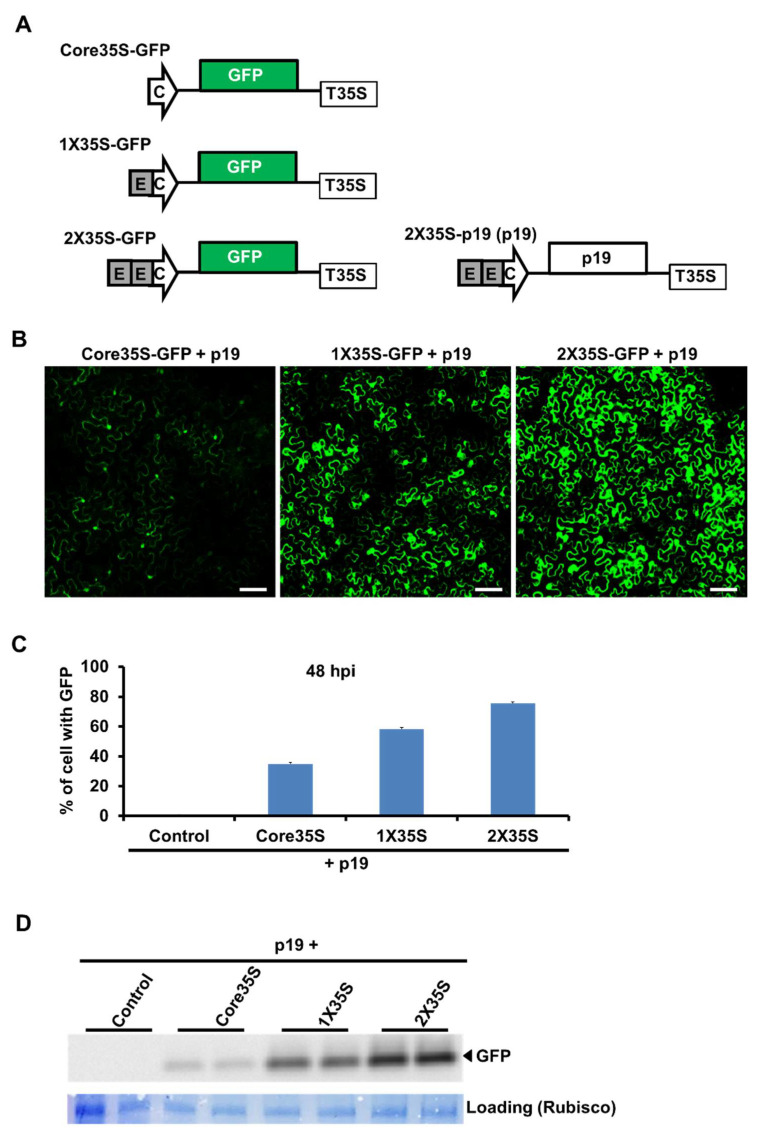
Variation of 35S promoter strengths in *N. benthamiana* plants determined by GFP expression. (**A**) Schematic representation of the different 35S promoter based GFP constructs. (**B**) Microscopic (confocal microscopy) images of *N. benthamiana* leaves infiltrated with the different 35S promoter-based GFP constructs with the p19 construct. The size bar = 100 μm. (**C**) Expression (%) of cell with GFP. Five same size pieces of leaves were taken from five different inoculated plants. One appropriate scanning field comprising 45 to 60 cells was chosen. The total % cells with GFP in this field were countered at 48 hpi. (**D**) Western blot analysis to determine the level of GFP expression with a GFP antibody. Coomassie blue-stained Rubisco large subunit (*RbcL*) is shown as a loading control. This experiment was conducted twice with similar results. Here, arrowed-C indicates Core35S promoter, gray-boxed-E indicates enhancer, boxed-p19 indicates a silencing suppressor obtained from tomato bushy stunt virus (TBSV), and boxed-T35S indicates the cauliflower mosaic virus 35S terminator.

To show the strength variation among 2X35S, 1X35S, and core35S promoters, we first generated a replication-independent GFP construct to the downstream of all three promoters as shown in Figure 1A. Twelve *Nicotiana benthamiana* plants were then agroinfiltrated with each of those constructs using *Agrobacterium*-mediated delivery. An *Agrobacterium* strain harboring a p19-expressing plasmid (a silencing suppressor of tomato bushy stunt virus (TBSV)) was co-delivered to enable high-level expression of GFP.

Confocal experiment results indicated that the level of GFP expression at 48 h post-infiltration (hpi) of these constructs was 2X35S>1X35S>core35S strength wise (Figure 1B). We also counted the GFP expressed cells at 48 hpi and identified around 80% cell expressed in 2X35S promoter-based construct, whereas there were only 40 and 60% of cells expressed with GFP in core35S and 1X35S constructs, respectively (Figure 1C). This result was also confirmed with Western blot hybridization assays with samples collected at 48 hpi. As shown in Figure 1D, the GFP expression level varies in the three versions of 35S promoters; 2X35S, 1X35S, and Core35S with the highest, medium, and lowest, respectively. These results indicated that 35S promoter variants have variable potency for the expression of target gene.

### 2.2. TCV-mCherry Replicon Is Variably Driven by the Three Promoters of Distinctive Strengths

**Figure 2:**
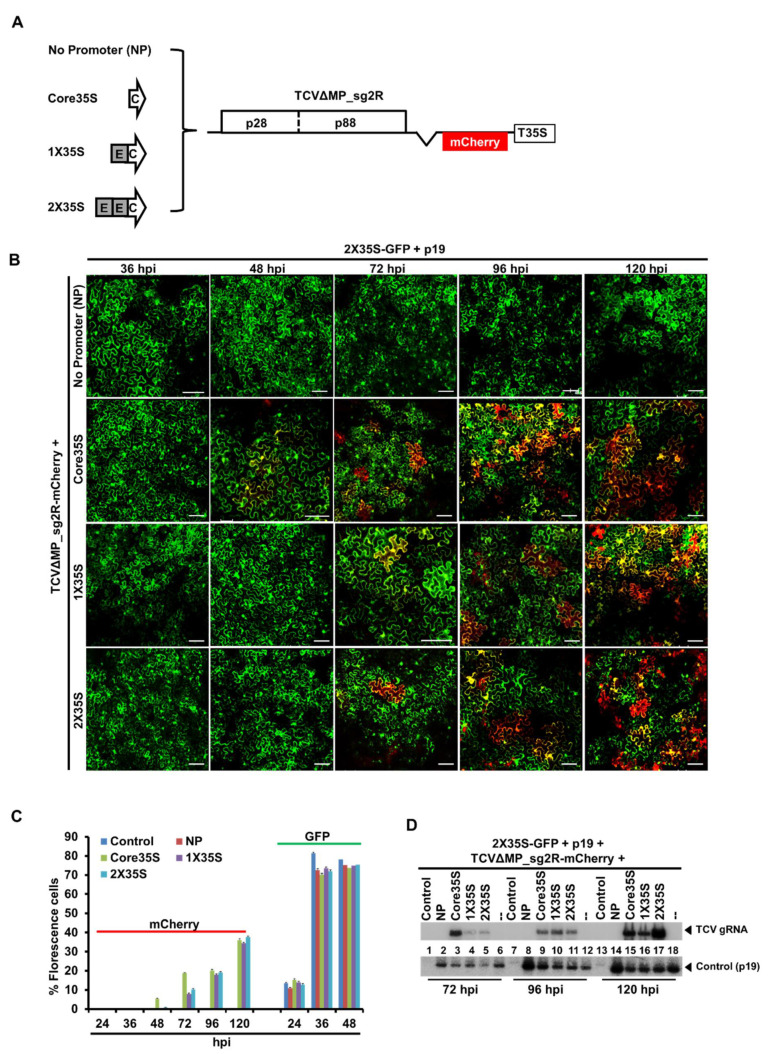
Replication efficiency of TCV-mCherry driven by the different strengths of the promoter variants. (**A**) Schematic representation of the four different 35S promoter-based TCVΔMP_sg2R-mCherry constructs; Core35STCVΔMP_sg2R-mCherry, 1X35STCVΔMP_sg2R-mCherry, 2X TCVΔMP_sg2R-mCherry, and NPTCVΔMP_sg2R-mCherry. (**B**) Microscopic (confocal microscopy) images of *N. benthamiana* leaves infiltrated with the different 35S promoter-based TCVΔMP_sg2R-mCherry with GFP and p19 constructs. The four different 35S promoter-based TCVΔMP_sg2R-mCherry constructs were delivered by agroinfiltration onto leaves of healthy *N. benthamiana* plants. A p19-expressing construct was included with all constructs to counteract RNA silencing-mediated degradation of RNA transcribed from infiltrated constructs and a GFP-expressing construct added to compare the timing difference of GFP and mCherry expression. The size bar = 100 μm. (**C**) Expression (%) of cell with GFP. Five same size pieces of leaves were taken from five different inoculated plants. One appropriate scanning field comprising 45 to 60 cells was chosen. The total % cells with GFP or mCherry in this field was countered at 36, 48, 72, 96, and 120 hpi, respectively. (**D**) Western blot analysis to determine the level of GFP expression with a GFP antibody. Coomassie blue stained Rubisco large subunit (RbcL) is shown as a loading control. This experiment was conducted twice with similar results. Here, arrowed-C indicates Core35S promoter, gray-boxed-E indicates enhancer, boxed-p28 indicates the TCV-encoded auxiliary replication protein p28, boxed-p88 indicates the TCV-encoded RNA-dependent RNA polymerase (RdRp) protein p88, and boxed-T35S indicates the cauliflower mosaic virus 35S terminator.

To test which promoter type will enable earlier replication of a TCV-mCherry replicon, we next inserted different versions of the 35S promoter sequence into the upstream region of TCV-mCherry cDNA (TCVΔMP_sg2R_mCherry2) through mutation analysis. As shown in Figure 2A, we constructed three different promoter variants with different strengths and a construct without having promoter region, namely, Core35STCVΔMP_sg2R-mCherry, 1X35STCVΔMP_sg2R-mCherry, 2X TCVΔMP_sg2R-mCherry, and NPTCVΔMP_sg2R-mCherry, respectively. These constructs were then delivered into *N. benthamiana* together with a P19, to counteract RNA silencing-mediated degradation of RNA transcribed from infiltrated constructs. A GFP-expressing construct (2X35S-GFP+p19) with the highest GFP expressions (Figure 1) was co-introduced to all treatments; so that we can compare the timing of florescence emergence from virus replication independent (2X35S-GFP+p19) and TCV replication dependent (TCVΔMP_sg2R-mCherry+NP/Core35S/1X35S/2X35S) constructs due to varying length of post-entry delays to initiate replication in the same cell. The green fluorescence from replication independent GFP construct appeared in most of the cells (about 70 to 80%) after 36 hpi. However, red fluorescence from replication dependent constructs with mCherry emerged only after 48 hpi in a few cells and gradually progressed afterwards (Figure 2B). Confocal microscopic observation was carried out at 36, 48, 72, 96, and 120 hpi and showed that core35S promoter enabled earlier replication of a TCV-mCherry replicon at 48 hpi, whereas 1X35S and 2X35S; replication starts at 72 hpi (Figure 2B). GFP/mCherry expressed cells were counted to compare among constructs for TCV-mCherry replicon abilities. It was revealed that about 9% of cells expressed with mCherry TCV replicon were driven by Core35S promoter at 48 hpi, whereas no mCherry cells were detected with other promoter-driven constructs at this time point. Core35S driven TCV-mCherry replication maintained its higher position up to 96 hpi; however, GFP, a replication independent construct, which was co-delivered with TCV variants were expressed in similar fashion (Figure 2C). Further Northern blot hybridization using TCV-specific probe showed that at 72 hpi, the highest accumulations of viral genomic RNA was detected in Core35S promoter-based variants compare to other promoters’ variants (Figure 2D). These results also proved that Core35S promoter-based construct enhances TCV-mCherry replicon at the initial stage. Other relatively strong promoter-based constructs were delayed at least 24 h after Core35S. All together, these results demonstrated that Core35S, a weaker promoter, enables earlier replication of a TCV-mCherry replicon than other stronger promoter-based TCV constructs.

### 2.3. Symptom Expression Comparison of the Wildtype TCV cDNA by 35S Promoter Variants with In Vitro Transcript

**Figure 3:**
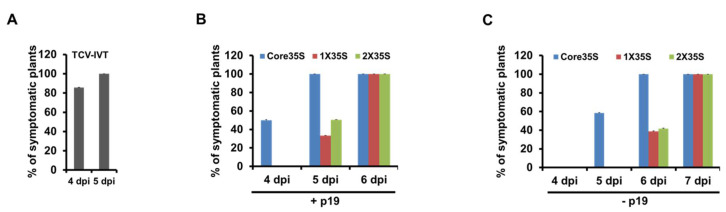
Symptom expression of the wildtype TCV on *N. benthamiana*. (**A**) Symptom expression (%) with rub inoculation method using in vitro transcript (IVT) of wtTCV. (**B**) Symptom expression (%) with agroinfiltrated delivery of three different promoter-based wt TCV constructs; Core35SwtTCV, 1X35SwtTCV, and 2X35SwtTCV, along with p19-expressing construct onto leaves of healthy *N. benthamiana* plants. (**C**) Symptom expression (%) with agroinfiltration method using three different promoter-based wt TCV constructs without p19.

To drive the expression of the wildtype TCV cDNA, we designed three constructs using the same set of promoters, namely, Core35SwtTCV, 1X35SwtTCV, and 2X35SwtTCV. Results showed that systemic symptoms appeared the earliest at 4 dpi in the Core35S-driven TCV cDNA (Figure 3B). To counteract RNA silencing-mediated degradation of RNA transcribed from infiltrated constructs, a p19-expressing construct was added with all treatments, which appeared one day earlier systemic symptoms than exclusion of the silencing suppressor (Figure 3B,C). Then we compared to see the difference of the infection methods between the Core35S promoter-driven TCV cDNA (along with p19) agroinfiltration and in vitro transcript (IVT) of wt TCV rub inoculation method. Results showed that systemic symptoms appeared almost at the same time on *N. benthamiana* using both TCV IVT rub inoculation and Core35S promoter-based TCV agro delivery method. However, more synchronous symptoms occurred using the IVT rub inoculation method.

## 3. Discussion

Understanding the role of promoters that drive the initiation of RNA virus replication from viral cDNA will be helpful to advance our knowledge of plant virus research. In the past, strong promoters were frequently used for generating infectious cDNA to maximize viral replication [21,22,23]. However, there is no direct evidence so far that compares among promoters (weaker/medium-strong/strong) for permitting more efficient initiation of virus replication in plants. The expression of a strong promoter may vary on genes of interest and its interaction with other factors [15,16]. In the current study, we used different versions of the 35S promoter-based TCV cDNA to investigate the effect of weaker promoters on viral replication in *N. benthamiana*.

Here, we first compared three versions of the 35S promoter for their ability to power the expression of a non-replicating green fluorescent protein (GFP) gene and it demonstrated that 2X35S and Core35S correlated with the highest and lowest GFP expression, respectively (Figure 1). These data suggest that three versions of 35S promoters do have different strengths. Our finding is consistent with a previous study by Wu et al. [24] that compared the strength of 2X35S and 1X35S promoters for MYB75 transient expression; it showed that the expression level driven by 2X35S was 6.6 times higher than 1X35S driven vectors. So, it is reasonable to think that the strong promoter 2X35S-driven TCV cDNA replication will be higher than the other weaker versions of the 35S promoter. For clarifying this possibility, we next analyzed the TCV-mCherry driven by different strengths of 35S promoters (2X35S/1X35S/Core35S/NP) and interestingly it showed that the exact opposite is true. Our findings demonstrated that Core35S, a relatively weaker promoter, enables higher and earlier replication of TCV-mCherry than the other stronger versions of 35S promoter (Figure 2). A recent study with a weak promoter tCUP1 (a fragment derived from the tobacco cryptic promoter) showed that the use of a weak promoter led to an increase in the expression of the foreign gene in transgenic rice [25]. They did not mention clearly the reasons behind the higher expression; however, Sanchez et al. [26] demonstrated that the stronger promoter enables higher accumulation of the specific protein that becomes a repressor at higher cellular concentration. A recent report by Zhang et al. [27] demonstrated that the strong 2X35S promoter-driven transcription of the 5′ proximal auxiliary replication protein p28, whether with C-terminal modifications with a double HA (2HA) tag, or a fluorescent protein (GFP or mCherry), or without any terminal modifications completely shut down the replication of TCV in the same cells. Another study by Zhang et al. [28] reported a dramatic reduction of TCV gRNA and sgRNA replicon upon higher accumulation of TCV-encoded RNA-dependent RNA polymerase (RdRp) p88 protein concentration, which supports the idea of suppression of TCV replication by abundance of its own protein. We further examined systemic movement using the same set of promoters to drive the expression of the wild type TCV cDNA. The results showed that Core35S-driven TCV cDNA initiates systemic symptoms the earliest than other stronger versions of 35S promoter, and inclusion of a p19-expressing construct appeared to accelerate a little (Figure 3). Our experiment with IVT of TCV also demonstrated that almost the same time is needed for systemic movement for IVT and Core35S methods (Figure 3). However, more synchronous occurrence of symptoms appeared in IVT rub inoculation method. Taken together, our findings showed that the relatively weaker Core35S promoter enables to initiate earlier TCV replication. The efficiency of the 35S promoter might be dependent on the virus. More detailed study is needed on other virus genomes to draw a broader conclusion on which would be a better choice for generating infectious cDNA clones of plant viruses.

## 4. Conclusions

In this study, we designed a weaker version of the 35S promoter and investigated its strength for foreign gene expression along with viral RNA replication. Results confirmed that the Core35S promoter is weaker than other versions of the 35S promoter; however, it enabled earlier replication of TCV-mCherry replicon. A strong promoter 2X35S driven initiation of TCV infection was delayed by at least 24 h than Core35S. A systemic infection of *N. benthamiana* leaves with wildtype TCV with 35S promoter variants also provided the similar delay. These findings suggested that a higher accumulation of a TCV protein by the stronger 2X35S promoter could be a repressor of TCV replication at higher cellular concentration. The Core35S promoter is likely to accumulate a smaller amount of TCV protein at the same time, which is helpful for avoiding repressor activity, and would be a better choice for generating infectious cDNA clones of TCV.

## 5. Materials and Methods

### 5.1. Plant Materials

Wild type *Nicotiana benthamiana* plants were used in this study. Plants were reared in a growth room with the temperature set at 22 °C in 15 h light and 9 h dark.

### 5.2. Plasmid Construction

We used PCR to produce variations of 35S promoter from the template pRTL-CMV2b and cloned into pAI101 at *Pst I* restriction site [29]. Then, the TCV-mCherry2 insert moved into the new pAI101 derivatives to create 2X35STCVΔMP_sg2R-mCherry2, 1X35S TCVΔMP_sg2R-mCherry2, as well as CoreTCVΔMP_sg2R-mCherry2 (Figure 2A, Figure S1). All constructs were sequenced to confirm their identities. The 2X35S-GFP and 2X35S-p19 constructs were described in earlier studies [13,27]. The other modified variants of GFP constructs, i.e., 1X35S-GFP and Core-GFP were built in this study (Figure 1, Figure S1). The Core35SwtTCV, 1X35SwtTCV, and 2X35SwtTCV were also created for symptom expression study of the wildtype TCV cDNA by 35S promoter variants according to the method described above (Figure 3, Figure S1). 

### 5.3. Agroinfiltration of N. benthamiana Plants

Wild type *N. benthamiana* leaves were agroinfiltrated on the first two true leaves. Each of those confirmed constructs used in this study (described in Section 5.2) were electroporated into *Agrobacterium tumefaciens* strain C58C1. Distinct combinations of *Agrobacterium* suspension carrying the constructs used in this study were agroinfiltrated as described previously by Qu et al. [13]. An *Agrobacterium* strain carrying p19-expressing construct was included with the respective experiments to counteract RNA silencing mediated mRNA degradation. Three plants (first two true leaves per plant) were used for each construct and the trial was conducted twice.

### 5.4. Infection of N. benthamiana Plants with In Vitro Transcripts

Using the TranscriptAid T7 High Yield Transcription Kit (Fermentas, Glen Burnie, MD, USA), in vitro transcripts of TCV were produced and purified according to the kit’s instruction with some modifications. The integrity of the transcripts was examined with agarose gel electrophoresis. For mixed infections, an equal amount (10 μg) of transcript RNA was withdrawn from each purified transcript and combined with each other to make the mixed inoculum. The mixed inoculum was further diluted to 10 ng/μL with an inoculation buffer containing 50 mM glycine, 30 mM K_2_HPO4, pH 9.2, 1% bentonite, and 1% celite. For mechanical inoculation of *N. benthamiana* leaves, 20 μL of these 10 ng/μL inoculums was spotted on each leaf and gently spread with a gloved finger. A total of six plants were used to inoculate (first two true leaves per plant) for each construct and in vitro transcript (IVT) of wt TCV. The trial was conducted twice.

### 5.5. RNA Blot Analysis

Total RNAs was extracted from the infiltrated leaves (first two true leaves) using the TRIsure reagents kit according to the manufacturer’s instructions and subjected to RNA blot analysis to detect TCV viral RNAs as described previously [13,29,30]. Probe was generated by end-labeling oligonucleotides complementary to the corresponding variant inserts with radioactive gamma 32P ATP and T4 polynucleotide kinase (Thermofisher Scientific, Waltham, MA, USA). The assay was carried out two times.

### 5.6. Western Blot Analysis

Protein extracts were prepared from agroinfiltrated leaves tissues as described previously by Cao et al. [31]. The anti-GFP antibody was purchased from Sigma-Aldrich (St. Louis, MO, USA). This experiment was conducted twice with duplicates.

### 5.7. Confocal Microscopy

Confocal microscopic observations were carried out using a Leica Confocal microscope (TCS SP5) available through Molecular and Cellular Imaging Center at the Ohio Agricultural Research and Development Center, The Ohio State University [29].

## Data Availability

The data presented in this study are available within the article or supplementary material.

## Supplementary Materials

The following are available online at https://www.mdpi.com/article/10.3390/plants10081700/s1, Figure S1: DNA sequence of the 35S promoter variants and 35S terminator.
