# Supplementary material for: A Core35S Promoter of Cauliflower Mosaic Virus Drives More Efficient Replication of Turnip Crinkle Virus
Source: Plants (Basel). 2021 Aug 18;10(8):1700. doi: 10.3390/plants10081700 (PMC8399983; doi:10.3390/plants10081700)
Supplement: Supplementary file 1 [file plants-10-01700-s001.zip › plants-1277914-supplementary.pdf]

```
ACATGGTGGAGCACGACACTCTCGTCTACTCCAAGAATATCAAAGATACAGTCTCAGAAGACCAGAGGGCTATTGAG
ACTTTTCAACAAAGGGTAATATCGGGAAACCTCCTCGGATTCCATTGCCCAGCTATCTGTCACTTCATCGAAAGGAC
AGTAGAAAAGGAAGATGGCTTCTACAAATGCCATCATTGCGATAAAGGAAAGGCTATCGTTCAAGAATGCCTCTAC
CGACAGTGGTCCCAAAGATGGACCCCCACCCACGAGGAACATCGTGGAAAAAGAAGACGTTCCAACCACGTCTTCA
AAGCAAGTGGATTGATGTGATAACATGGTGGAGCACGACACTCTCGTCTACTCCAAGAATATCAAAGATACAGTCTC
AGAAGACCAGAGGGCTATTGAGACTTTCAACAAAGGGTAATATCGGGAAACCTCCTCGGATTCCATTGCCCAGCTAT
CTGTCACTTCATCGAAAGGACAGTAGAAAAGGAAGATGGCTTCTACAAATGCCATCATTGCGATAAAGGAAAGGCTA
TCGTTCAAGAATGCCTCTACCGACAGTGGTCCCAAAGATGGACCCCCACCCACGAGGAACATCGTGGAAAAAGAAGA
CGTTCCAACCACGTCTTCAAAGCAAGTGGATTGATGTGATATCTCCACTGACGTAAGGGATGACGCACAATCCCACT
ATCCTTCGCAAGACCCTTCCTCTATATAAGGAAGTTCATTTCATTTGGAGAGG
```

GOI: mGFP5/p19/ TCVΔMP_sg2R-mCherry2/wtTCV

```
TCTAGAGTCCGCAAAAATCACCAGTCTCTCTCTACAAATCTATCTCTCTCTATTTTTCTCCAGAATAATGTGTGAGT
AGTTCCCAGATAAGGGAATTAGGGTTCTTATAGGGTTTCGCTCATGTGTTGAGCATATAAGAAACCCTTAGTATGTA
TTTGTATTTGTAAAATACTTCTATCAATAAAATTTCTAATTCCTAAAACCAAAATCCAGTGA
```

Yellow: The duplicated enhancer of the 35S promoter (327 nt)

Brown: The enhancer of the 35S promoter (327 nt)

Red: The Core35S promoter (89 nt)

Gray: The CaMV 35S terminator

Green: Genes of intertest inserted downstream of the promoter and upstream of the terminator

Supplementary Figure S1: DNA sequence of the 35S promoter variants and 35S terminator. The 2X35S promoter sequence has two identical copies of 35S region and the core region totaling 746bp (yellow + brown + red), while the 1X35S promoter sequence is 416bp harboring one copy of 35S with the core region (brown + red), and the length of Core35S promoter is 89bp (red). The genes of interest were inserted in between the 35S promoter variants and terminator (green). The blue box is showing the P35S-GOI-T35S (35S promoter variants-gene of intertest-35S terminator) and the red box is indicating the abbreviations of different color used in this figure.
